# Supplementary material for: Resolving the full spectrum of human genome variation using Linked-Reads
Source: Genome Res. 2019 Apr;29(4):635–45. doi: 10.1101/gr.234443.118 (PMC6442396; doi:10.1101/gr.234443.118)
Supplement: Supplemental Material [file supp_gr.234443.118_Supplemental_longranger-2.2.zip › longranger-2.2.tar.bz2!/lr2.2-public/lib/stan/cmdstan/stan/doc/api/html/bfgs_8hpp.html]

Stan: src/stan/optimization/bfgs.hpp File Reference


|  |  |
| --- | --- |
|  | Stan  2.5.0  probability, sampling & optimization |


- Main Page
- Related Pages
- Namespaces
- Classes
- Files

- File List
- File Members

All Classes Namespaces Files Functions Variables Typedefs Enumerations Enumerator Friends Macros Pages

- src
- stan
- optimization

Classes |
Namespaces |
Enumerations |
Functions

bfgs.hpp File Reference

`#include <cmath>`  
`#include <cstdlib>`  
`#include <string>`  
`#include <limits>`  
`#include <boost/math/special_functions/fpclassify.hpp>`  
`#include <stan/math/matrix/Eigen.hpp>`  
`#include <stan/math/matrix/meta/index_type.hpp>`  
`#include <stan/model/util.hpp>`  
`#include <stan/optimization/bfgs_linesearch.hpp>`  
`#include <stan/optimization/bfgs_update.hpp>`  
`#include <stan/optimization/lbfgs_update.hpp>`

Go to the source code of this file.

|  |  |
| --- | --- |
| Classes | |
| class | stan::optimization::ConvergenceOptions< Scalar > |
|  | |
| class | stan::optimization::LSOptions< Scalar > |
|  | |
| class | stan::optimization::BFGSMinimizer< FunctorType, QNUpdateType, Scalar, DimAtCompile > |
|  | |
| class | stan::optimization::ModelAdaptor< M > |
|  | |
| class | stan::optimization::BFGSLineSearch< M, QNUpdateType, Scalar, DimAtCompile > |
|  | |

|  |  |
| --- | --- |
| Namespaces | |
|  | stan |
|  | Probability, optimization and sampling library. |
|  | |
|  | stan::optimization |
|  | |

|  |  |
| --- | --- |
| Enumerations | |
| enum | stan::optimization::TerminationCondition {     stan::optimization::TERM\_SUCCESS = 0, stan::optimization::TERM\_ABSX = 10, stan::optimization::TERM\_ABSF = 20, stan::optimization::TERM\_RELF = 21,     stan::optimization::TERM\_ABSGRAD = 30, stan::optimization::TERM\_RELGRAD = 31, stan::optimization::TERM\_MAXIT = 40, stan::optimization::TERM\_LSFAIL = -1   } |
|  | |

|  |  |
| --- | --- |
| Functions | |
| template<typename M > | |
| double | stan::optimization::lp\_no\_jacobian (const M &model, std::vector< double > &params\_r, std::vector< int > &params\_i, std::ostream \*o=0) |
|  | |


---

[ Stan Home Page ]

*© 2011–2014,
Stan Development Team.*
